# Health Impact Assessment (HIA) of a Fluvial Environment Recovery Project in a Medium-Sized Spanish Town

**DOI:** 10.3390/ijerph17051484

**Published:** 2020-02-25

**Authors:** Cristina Casajuana Kögel, Tània Rodríguez Peña, Isabel Sánchez, Montserrat Tobella, José Alonso López, Fernando Girón Espot, Francesc Pedrol Claramunt, Gemma Rabal, Angelina González Viana

**Affiliations:** 1 Public Health Agency of Catalonia, Government of Catalonia, 08005 Barcelona, Spain; cristina.casajuana@gencat.cat (C.C.K.); trodriguezp@catsalut.cat (T.R.P.); fgiron@gencat.cat (F.G.E.); fpedrol@gencat.cat (F.P.C.); 2 Ajuntament de Sant Andreu de la Barca, 08740 Barcelona, Spain; isanchez@sabarca.cat (I.S.); mtobella@sabarca.cat (M.T.); 3 Àrea Metropolitana de Barcelona, 08040 Barcelona, Spain; jalonso@amb.cat; 4 Universitat Pompeu Fabra, 08003 Barcelona, Spain

**Keywords:** Health in All Policies, Health Impact Assessment, social determinants of health, HIA best practices

## Abstract

Introduction: The Interdepartamental Public Health Plan of Catalonia (2014) seeks to enforce Health in All Policies (HiAP) at the regional and local levels. Within this context, the City Council of Sant Andreu de la Barca (SAB), the Metropolitan Area of Barcelona (MAB), and the Public Health Agency of Catalonia started a Health Impact Assessment (HIA) of an urbanistic redesign of the Llobregat fluvial area in SAB, the results of which are presented in this paper. Methodology: In 2018, after a HIA screening, a prospective nonquantitative HIA was conducted. Politicians, professionals, and citizens participated in identifying potential impacts. Impacts were prioritized and linked to health determinants, scientific evidence, and potentially affected social groups. Afterwards, recommendations were formulated in order to improve the health impacts of the project. Finally, indicators were selected to evaluate HIA implementation. Results: The HIA was successfully implemented with the participation of technicians and citizens of SAB. The health impacts identified were mainly related to environmental, public safety, lifestyle, socioeconomic, and political contexts. Ten recommendations were defined to minimize the potential negative health impacts of the project, with six of them directly included and only one dismissed due to incompatibility. Conclusion: A HIA was successfully carried out in the medium-sized town of Catalonia, promoting Health in all Policies at a local level and improving health impacts of an urbanistic project.

## 1. Introduction

Approximately 80% of the determinants of health are outside the health system [1]. Policies, programs, or projects that affect the environmental, social, and economic conditions in which people live have important effects on health [2,3,4]. Intersectoral action thus becomes key to improving population health. Tools are required to support decision-making and to facilitate incorporation of Health in All Policies (HiAP) approaches, especially in the context of little experience and scarce application of HiAP. 

Health Impact Assessment (HIA) is a combination of procedures, methods, and tools by which a policy, program, or project is assessed in relation to its potential effects on the health of a population, taking into account the distribution of these effects [5]. It provides a vision of integral health, which includes social determinants of health, equity, intersectional work, and participation. The HIA helps to implement the vision of HiAP by conveying the incorporation of health and equity in the design and implementation of intersectoral policies. It provides transparency for decision-making about non-health-related policies, emphasizes equity, and reduces health inequalities in planning and the political agenda [6]. 

The Public Health Law of Catalonia (18/2009) [7] and Spain (33/2011) [8] introduced the HIA as a public health mandate to be performed on policies or programs affecting determinants of health. HIA implementation is a responsibility of public health professionals. The Catalan Interdepartmental Plan of Public Health [9] also recommends other government departments to perform HIA and has developed a screening tool called Test Salut [10] to facilitate it. Test Salut allows a simplified HIA to be carried out in order to (1) evaluate policies or interventions before approval; (2) help estimate potential impacts of the intervention on the health of the population; and (3) make recommendations to minimize the possible negative impacts and maximize the positive ones, taking into account their distribution in the different social groups.

In 2018, the City Council of Sant Andreu de la Barca (henceforth referred to as Sant Andreu), in coordination with Barcelona Metropolitan Area (AMB), approved a project to redesign the Llobregat’s fluvial area as it passes through Sant Andreu. The aim of the project was to improve population access to the fluvial area as well as to regenerate its wildlife, with the final aim of improving citizens’ health and well-being. At the initiative of the City Council of Sant Andreu, it was decided that a HIA would be implemented with the methodological support of the Public Health Agency of Catalonia (ASPCAT). 

Although other HIA have been conducted in our context [11,12,13,14], this was the first in which the ASPCAT was involved as part of its tasks. It was an opportunity to review the roles and capacities of public health professionals regarding the development of a HIA. The aim of this paper is to present the results of the implementation of the HIA on the urban redesign of the Llobregat fluvial area in Sant Andreu, Spain.

## 2. Methods

A prospective nonquantitative HIA was performed of the urban redesign of the Llobregat fluvial area as it passes through the municipality of Sant Andreu. The HIA considered one scenario, and its specific objectives were as follows: (1) assess potential positive and negative health impacts of the project; (2) estimate the distribution regarding age, sex, and socioeconomic class of potential health impacts; and (3) elaborate recommendations to propose changes and improvements.

Two working groups were established. First, a steering group was formed that consisted of the mayor, environment and public health area councilors, the APSCAT’s area manager, and the architect in charge of the project from the AMB. Second, an evaluation group was formed consisting of four technicians from the city council (public health area), the AMB, and the ASPCAT.

The HIA followed the six recommended steps of screening, scoping, assessment, recommendations, reporting, and monitoring and evaluation [15].

### 2.1. HIA Procedure

#### 2.1.1. Screening

Although the intervention was opportunistic, the steering group carried out the screening phase to confirm the relevance of implementing a HIA. The main criteria were as follows: (1) possibility of implementing project modifications derived from the HIA; (2) possibility of potential impacts on health from the project that should be measured and monitored; and (3) political will and resources to carry out the task. 

#### 2.1.2. Scoping

The evaluation group designed and planned the scope of the HIA. 

#### 2.1.3. Appraisal

*Analysis of the Intervention*: The AMB technicians explained to the steering and evaluating groups different elements of the intervention, which were analyzed in a comprehensive manner. Furthermore, a visit was done to the project area.

*Population Characterization*: Data were collected from the 2017 Health Report of general practitioners and the Municipal Health Plan (2013–2018) [16].

*Data Collection*: An experts’ meeting with technical personnel was held to assess the potential effects of the project. Primary health care professionals and the police were interviewed as they could not assist in the meeting, and their opinions were incorporated. Moreover, a discussion group was formed with the general population (convenience sample) and associations. All three were facilitated by public health professionals with previous experience in qualitative methodology. Test Salut was used as a framework and as a script for the focus groups and interviews.

*Impact Analysis*: The potential impacts on health, health determinants, and health equity that had been identified were verified through a literature review. The literature search was based on the World Health Organization report on green spaces and health [17], and ad-hoc searches were conducted when necessary. 

*Impact Prioritization*: For the prioritization, health impacts were selected by their relevance, such as being mentioned by citizens, by the exiting magnitude of scientific evidence, and by the feasibility of implementing recommended actions.

#### 2.1.4. Recommendations

Based on the information collected, the evaluation group issued a list of recommendations. These were presented to the steering group, which decided which ones would be incorporated in the project. Once agreed, all participants (professionals, neighbors, and key informants) were invited to a final session, where the recommendations were explained to the public and the reasons not to incorporate them were justified. 

#### 2.1.5. Reporting

A report to present the different steps and results was prepared by ASPCAT technicians. The document (in Catalan) was shared with all stakeholders. Once agreed, the final version was uploaded in institutional websites.

#### 2.1.6. Monitoring and Evaluation

The HIA results were presented to the community. A plan to monitor and evaluate the HIA was devised. 

## 3. Results

The steering and evaluation groups were created in September 2017, and the HIA was carried out following the steps below. 

### 3.1. Screening

Performing a HIA was considered relevant. Recommended modifications derived from the HIA could be implemented, potential impacts on health were expected, and there was political will and resources to carry out the task.

### 3.2. Scoping

A prospective in-depth and “nonquantitative” HIA was planned to be carried out between September 2017 and May 2018. 

### 3.3. Appraisal

#### 3.3.1. Analysis of the Intervention

The project included a series of actions in the fluvial environment to improve accessibility to pedestrian and cyclist pathways, signaling, and biodiversity.

#### 3.3.2. Population Characterization

Of the 27,303 inhabitants (2017 census data) [18], 50.2% were women. In comparison to Catalonia, Sant Andreu had a younger population (children, 19.7% in Sant Andreu versus 15.6% in Catalonia; older people, 14.1% in Sant Andreu versus 18.5% in Catalonia) and only 10.7% immigrants (with Moroccan origin being the most common at 37.2%). Household disposable income per capita was lower than Catalonia’s average (€13,356 vs. €16,367), and the educational level was lower (only 1.2% had higher education versus 21.5% in Barcelona). The main chronic health problems attended by primary health care professionals were lipid metabolism disorders, hypertension, and anxiety disorders, which were quite similar to the results in Catalonia. 

#### 3.3.3. Data Collection

The experts’ meeting comprised six technicians from the city council (areas of culture, environment, public health, and public transport), the AMB architect in charge of the project, two representatives from the ASPCAT, and two representatives from the provincial government. The citizens’ discussion group was attended by 16 people (11 men, average age 55 years) from nine municipality associations. Table 1 shows the potential effects that were identified regarding health, health determinants, and equity. In general, participants agreed on the benefits of the project but also showed their concern for adequate maintenance once implemented. 

#### 3.3.4. Impact Analysis

A nonsystematic literature review (N = 41) was done to corroborate the potential impacts that had been identified or to find new ones (see Table 2). 

#### 3.3.5. Prioritization of Impacts

Table 2 describes evidence of the effects on health, health determinants, and health equity that were identified. Health impacts were characterized by their direction, severity, magnitude, likelihood, and distribution within the population. Finally, 11 determinants were identified, four of them with a negative impact on health and seven with a positive impact. 

### 3.4. Recommendations

A list of recommendations was drafted and is summarized in Table 3. There were six recommendations to minimize the potential negative impacts and 17 to maximize the possible positive impacts.

### 3.5. Reporting

The HIA results and selected recommendations were returned to the citizens and technicians that had participated in the appraisal. A report was also published in Catalan describing all the steps and results; this is accessible from the municipality and ASPCAT websites [56].

### 3.6. Monitoring and Evaluation

The steering group responded positively to all the recommendations. However, five of them were not approved. The reasons for not approving were as follows: (1) not feasible (controlling potential increase in housing prices); (2) not compatible with the project (to illuminate the pathway at night, which was harmful for the fauna); (3) not prioritized (solar panels, video surveillance, and bike parking). 

The construction phase of the project began in January 2019. The evaluation group selected a list of indicators to assess HIA implementation (Table 4) and will be in charge of monitoring and evaluating it. Moreover, a brief survey was designed in order to collect data on citizens’ perception of health as well as potential changes in the main motivations to use the remodeled fluvial area. This survey, administered before the execution of the project (January 2019), is expected to be administered after the execution of the project (January 2020) using an opportunistic sample recruited in the area. 

## 4. Discussion

The HIA on a fluvial area regeneration was successfully carried out in a medium-sized Spanish town using the participatory methodology with the collaboration of different stakeholders and a special focus on equity. This HIA provided solid evidence-based information from qualitative and quantitative sources on the potential negative impacts and the potential benefits of the project. Impacts were prioritized, and a set of recommendations were issued in order to minimize the negative impacts and maximize the benefits. A HIA evaluation is in the process using a pool of indicators. 

The potentially negative health impacts identified were related to gentrification (due to increase in housing prices), allergies caused by introduction of specific vegetation in the area, lack of security, and risky behaviors. In contrast, the potentially positive impacts identified by our participants were general improvement of the municipality conditions, increase in green areas and sustainable mobility, reinforced security and promotion of physical activity, and self-care and healthy leisure. These results are in line with the results of other HIAs of urban projects in our context. For example, Bacigalupe et al. identified lack of security and risky behaviors as potential negative impacts on health of an urban project in the city of Bilbao, Spain [11]. Other HIAs have also reported gentrification as a consequence of urbanization projects, with potentially negative health effects, especially for the most vulnerable groups [13,57]. The positive impacts identified in this work are also similar to European projects like Blue Health, which especially emphasizes the benefits of riversides in increasing physical activity [58]. A recent study by Vert et al. estimated that promoting riversides for physical activity would improve social cohesion and social interaction. The authors went one step further by also estimating health-related economic benefits. The prospective HIA of Sant Andreu requested by the municipality prior to the execution of the project adds new knowledge to the ones reported in our context and could allow future comparison and analysis to identify which aspects contribute the most to health benefits [13,59]. 

Implementing the HIA in this context was relevant for several reasons. First, this was the first HIA carried out by ASPCAT, proving a valuable learning process for professionals who had only received theoretical HIA training. This experience brings HIA closer to being incorporated in the ASPCAT services’ catalog. Second, AMB architects and non-health-related council technicians valued the HIA positively as it improved the project from a health perspective and articulated a participatory process. The alliances generated would allow HIA to be implemented in other projects in the near future. Third, the ability to implement a HIA within the approval process of an urban project and in time to issue recommendations is a turning point for Catalonia as it shows how HIA can be a sustainable tool that adds value and transparency to projects. All these aspects are important in a context of scarce application of tools for HiAP. 

This paper reports all the phases of the HIA, an exercise of transparency that, in a context of unusual HIA applications, would become helpful for replication. Moreover, our results may be useful to raise awareness about the health impacts of similar projects. Monitoring is essential in order to accumulate evidence on the final health impacts of the project [59] and to facilitate evidence-based policy [60].

A selection of indicators for monitoring and evaluation of HIA have been included. These final steps are crucial to assess the HIA, as affirmed by Venegas-Sánchez et al. [13]. Monitoring the inclusion of the recommendations and defining a set of indicators are necessary to measure the final impact of the project. The indicators will be collected by different agents, which will help to maintain participation and interaction between the actors.

Some of the recommendations were not prioritized for the initial phase of the project. For example, the recommendation on illumination of the fluvial area pathways at night was rejected as it clashed with the objective of protecting the river’s fauna. Nevertheless, there was a commitment to monitor security and implement actions if necessary.

Implications of the HIA will be seen in the short, medium, and long terms. In the short term, it is foreseeable that the possible negative health impacts of the intervention will be minimized and that awareness of the effects of similar interventions on the determinants of health and health equity will increase. As the monitoring of the effects of the intervention takes place, medium-term effects should be apparent. One of the expected effects is an increase in citizen empowerment as they might feel more qualified to take part in local decisions that will ultimately affect them. 

In addition, there can be an increase in political responsibility and transparency, especially if they know whether or not the HIA recommendations have been implemented. Finally, in the long term, the impact should be reflected in basic health indicators that have been selected to monitor the impact of HIA. 

This study has some potential limitations. One of them is the empirical basis on which some impacts were estimated, which were also limited by the available evidence that came from very different contexts in most cases [61,62,63]. Most HIAs are based on empirically based impacts [64]. However, in this case, information collected in the qualitative phase complemented the evidence and helped to adapt the potential impacts and recommendations to Sant Andreu. In addition, some citizen groups were not represented in the qualitative phase, such as the youth. This is a common limitation as young people are difficult to engage in these kinds of projects [65]. In order to provide their point of view, the city council’s area manager responsible for culture and youth was invited to participate in the process. 

This study also has some potential strengths. Test Salut proved to be useful to facilitate the technicians’ discussion group and the participatory process. The timings of the HIA were optimal in order to be able to incorporate proposals. 

This HIA is part of a wider local strategy of Health in All Policies. Sant Andreu is part of the WHO Healthy Cities network, a long-term international development initiative that aims to place health on the agendas of decision-makers and to promote local strategies for health protection and sustainable development [66]. Our experience has been that it has helped to engage actors. Finally, HIA has helped to involve citizens in the design of the project while introducing them to the concept of Health in All Policies. Citizen participation and engagement increases empowerment [67,68] and, at the same time, improves transparency and democracy [69]. 

In order to enforce HiAP, HIA should be a service provided by public health agencies and professionals to municipalities and other political actors. This requires capacity building and resource allocation. Other challenges include systematizing HIA processes and providing evidence in order to facilitate its implementation.

Another pending issue is to regulate the use of HIA in Catalonia, which is currently a recommendation, so that the willingness depends on municipalities, project managers, or politicians’ motivation. This is not the case in Andalucía, where HIA was recently made compulsory for certain urban projects [70].

## 5. Conclusions

In Spain, HIA of non-health-related interventions are still infrequent. Reports like ours show that performing a HIA before the implementation of an urban regeneration project has great benefits. It sheds light on the health impacts of non-health-related interventions, incorporates participation and intersectoriality to decision-making, and helps to minimize potential effects of the intervention while helping to justify the investment of resources in social determinants. Moreover, implementation of the HIA was beneficial on its own as it facilitated mutual learning and fostered consensus and synergies by working in a transversal, intersectoral, and participatory manner. 

However, up to now, the willingness of a municipality still plays a crucial role in applying HiAP and enrolling the participation of citizens and entities. Therefore, one main challenge for public health agencies will be to encourage politicians to work in line with the HiAP principles. Public health actors should prioritize giving support to HIA and plan to invest in capacity building and resource allocation.

## Figures and Tables

**Table 1:** Potential effect on health, health determinants, and heath equity.

THE POLICY WILL HAVE EFFECT ON	Affected Population	Description of the Health Determinants	Possible Impact on Health
**Intermediate Determinants**			
**Material Conditions**			
Housing conditions	Entire population, especially Solana neighbors	Housing price increase Displacement from nearby neighborhood of poor and vulnerable groups Reduction of consumption capacity	Increased anxiety and stress Risk of increased mortality from all causes
Conditions of the neighborhood/area	Entire population, especially Solana neighbors	Reduction of pollution Improvement of acoustic comfort Increased sense of security in the area	Decreased feelings of stress, insomnia, and number of injuries (if robberies decrease)
Environment	Entire population	Reduction in pollution levels and improvement of air quality Increase in green areas Risk of environmental deterioration	Decrease in respiratory, cardiovascular, cerebrovascular, and metabolic diseases Decrease in mortality Increased life expectancy and quality of life Improvement of industry and neighborhood relationships
Access to services and basic goods	Entire population	Improved access to health and social services, etc.	Improvement of access to health system, increase in prevention and health promotion
Infrastructure of public transport and mobility	Entire population, workers	Increased sustainable mobility; cycling and active transportation Decreased road traffic	Increase in physical activity levels
**Psychosocial Factors**			
Public safety	Entire population, women and elderly	Better lighting → decreased insecurity feeling	Decreased crime-related stress; reduction of social isolation and increase in physical activity, social networks, etc.
Support and networks	Entire population	Increased social cohesion Increased sense of belonging Increase in citizen participation; empowerment of citizens/of citizenship	Improved mental health (increased self-esteem, less depression and anxiety) Reduction in cardiovascular diseases Decrease in mental illness, suicide, etc.
**Lifestyles**			
Physical activity	Entire population	Increased willingness to take care of oneself and take responsibility for their health	Increase in healthy behaviors in terms of food, physical exercise, tobacco, alcohol, and other drugs due to domino effect
Physical activity	Entire population	Increased levels of physical activity	Improvement of mental health Reduced risk of cardiovascular diseases, obesity, type 2 diabetes, colorectal and breast cancer, mortality, dementia, depression, etc. Decrease in the incidence of obesity and overweight in children and adolescents
Tobacco	Entire population	Increased self-care and responsibility over own health	Increased healthy behaviors in terms of food, physical exercise, and drug use due to a domino effect
Consumption of alcohol	Young people and adolescents	Risk of using green space for binge drinking	Increase in drugs consumption Increase in binge drinking
Sexual practices	Young people and adolescents	Risk of increased sexually risky behaviors	Increase in unwanted pregnancies Increase in sexually transmitted diseases (STD)
**Health System**			
Accessibility	Entire population, especially elderly people	Easier access to public services	Improvement of accessibility indicators to health services, preventive practices, etc. Improved self-perceived health
**Structural Determinants**			
**Social Cohesion**			
Social cohesion	Entire population, especially most vulnerable groups	Increased social cohesion Reduction of inequalities	Improvement of emotional well-being and reduction of mental health disease and mortality
**Socioeconomic and Political Context**			
Governance	Entire population	Feeling of control over decisions taken in municipality	Improved mental health and self esteem
Labor policies	Entire population	Improved access to industrial area (biking, walking) Decreased traffic to industrial area Decreased pollution level	Increased physical activity levelsDecrease in respiratory diseases

**Table 2:** Impact analysis results.

Description of the Health Determinant	Possible Impact on Health	Type	Evidence Found	Social Inequality in Health
**General improvement of conditions in the municipality** - Increased sense of belonging to the municipality - Increased citizen participation and empowerment - Increased feeling among the population of having control over the decisions taken in the municipality	Improvement of mental health; increase in self-esteem, less depression, anxiety, decrease in mental illnesses, suicides, etc. Reduction of cardiovascular diseases	+	Focus groups Bibliography [19,20,21,22,23]	Entire population
**Improvements on the environment due to the increase in green areas** Reduction of air pollution, river water, and noise perception Reduction of the environmental temperature	Decrease in respiratory diseases, etc. Decreased feelings of stress and insomnia Improvement of social cohesion and associative fabric	+	Focus groups Bibliography [24,25,26,27,28]	Entire population
**Reforestation of native species, such as poplars**	Risk of increased allergy episodes	-	Focus groups	Entire population, especially those most sensitive to allergens
**Risk of rapid deterioration of the environment due to poor maintenance of the area**	Increased conflict, vandalism, and risky practices by young people Loss of purchasing power of the neighbourhood of La Solana or the municipality Decreased mental health and well-being due to decreased physical activity	-	Focus group (neighbours) Bibliography [26]	Entire population, especially most vulnerable groups
**Risk of housing price increase** Risk of displacement from the area Increase in inequalities	Increased anxiety, stress Loss of social network Risk of increased mortality	-	Focus groups Bibliography [29,30,31]	Poor, women, children, the elderly, and members of racial/ethnic minority groups
**Improvement of sustainable mobility** Increase in active mobility (walking, cycling, etc.) for leisure and transportation to work/school Decreased traffic and pollution	Increased physical activity Decrease in injuries due to traffic Decrease in pollution	+	Focus groups Bibliography [32,33,34]	Poor, women, children, the elderly, and members of racial/ethnic minority groups
**Improved accessibility to the industrial area**	Increase in physical activityDecrease in respiratory diseasesIncrease in occupation	+	Focus groups Bibliography [35,36]	Entire population, especially the most vulnerable
**Improvement of security** (improvement of lighting and aesthetics)	Increased physical activity Improvement of mental health and reduction of stress associated with less crime and vandalism Reduction of social isolation Improvement of social cohesion and associative networks	+	Focus groups Bibliography [37]	Women, elderly people, and children
**Increase in the practice of physical activity** (increased access and improved environment)	Improvement of mental health Reduction of the risk of cardiovascular diseases, obesity, type 2 diabetes, and colorectal cancer Decrease in the incidence of obesity and overweight in children and adolescents The benefits of physical activity outweigh the possible risks of doing it in an environment with the presence of environmental pollutants	+	Focus groups Bibliography [38,39,40,41,42]	Entire population, poor Less benefit to single mums and caretakers (specially women)
**Increase in self-care** (increased self-care and responsibility for a healthier life)	Increase in healthy behaviours in terms of nutrition and addictions (alcohol, drugs, tobacco, screens) by a domino effect	+	Focus group (professionals) Bibliography [43,44,45]	Entire population, people with cardiovascular risk factors
**Increase in risky practices** (use of green space for risky behaviours)	Increase in alcohol consumption (binge drinking and drinking outdoors), injuries and violence, sexually risky behaviours, unintended pregnancies, and STDs	-	Focus groups Bibliography [46,47,48,49,50]	Adolescents and youth
**Increase in the use of spaces near the riverside for healthy leisure**	Increase in physical activity Active and healthy family Improvement of cohesion and social network	+	Focus Groups Bibliography [51,52]	Poor and families with children
**Promotion of social cohesion and reduction of inequalities**	Improvement of emotional well-being Diminution of mild mental health pathologies Prevention of loneliness	+	Bibliography [53,54,55]	Entire population Elderly people

**Table 3:** Prioritized impacts and related recommendations to minimize/maximize health impacts.

Identified Health Determinant	Recommendation	
**Negative Impacts**	**Recommendations to Minimize Health Impact**	**Recommendation Accepted**
Risk of increased allergy episodes due to reforestation with native species	Take action to avoid increase in allergies	Yes
Risk of project deterioration if the maintenance is poor	Implement measures to favor the maintenance of regenerated area; allocation of budget, establish alerts line/mail to report damages, promote voluntary maintenance activities	Yes
Risk of neighborhood gentrification and housing price increase	Take action to avoid increase in housing prices	No
Increase in risky practices (alcohol, tobacco, other drugs, sex, extreme sports, etc.)	Promotion of healthy activities and active leisure	Yes
	Avoid isolated and inconspicuous spaces	Yes
	Establish and disseminate regulations for the use of the space	Yes
**Positive Impacts**	**Recommendations to Maximize Health Impact**	
Promotion of social cohesion, participation, and empowerment	Actions to raise awareness about the potential of the new green areas on cultural, educational, health, and social cohesion for the community	Yes
	Promote educational programs in schools in the municipality to give value to the natural heritage	Yes
	Establish an area for birdwatching and informative panels about the local wildlife, in collaboration with the Tourism and Culture Department	Yes
Improvements in the environment due to the increase in green areas	Use of solar panels for lightening the accesses (citizens’ focus groups)	No
	Maintain and increase work with local industries to reduce their polluting impact	Yes
	Informative campaign on local pollution levels to diminish feelings/rumors of high/hazardous levels	Yes
Improvement of sustainable mobility, especially considering accessibility to the remodeled area and to the industrial area	Promote active transportation, with campaigns targeting industrial workers	Yes
	Possibility of incentives to companies promoting active transportation among workers	Yes
	Implement traffic safety measures in the area to avoid accidents	Yes
Improved security at the remodeled area	Improve security, especially during dark hours (lightning accesses)	Yes
	Ensure correct lighting of pathways at night	No
	Install video surveillance cameras in tunnels	No
	Install a sensor that counts access to evaluate the use of the area	Yes
	Install urban furniture to avoid entry of unauthorized vehicles to the fluvial area	Yes
Increase in physical activity levels	Ensure good accessibility in and to the area, avoid architectural barriers, and improve access for persons with functional diversity; also consider aesthetics	Yes
Increase in self-care	Organization of health promotion activities in the remodeled area	Yes
Increase in the use of spaces near the fluvial area for healthy leisure	Install urban furniture to park bicycles	No

**Table 4:** Health Impact Assessment (HIA) evaluation: selected indicators and source of information.

Indicator	Source
Number of participatory events in the remodelled area by year (for example, guided walks)	City council
Number of community programs initiated by the city council	City council
Number of social prescriptions made by primary health based on the use of the remodelled area	ASPCAT
Prevalence of cardiovascular diseases, pulmonary diseases, overweight, and obesity of all the population of Sant Andreu de la Barca (SAB) (segregated by age and gender)	AQUAS
Prevalence of anxiety disorders and distress (segregated by age and gender)	AQUAS
Percentage of tobacco smokers (segregated by age and gender)	AQUAS
Percentage of risky alcohol consumption (segregated by age and gender)	AQUAS
Percentage of illicit drug consumption (segregated by age and gender)	AQUAS
Evolution of main environmental indicators (including sound map)	City council
Kilometres of urban green	City council
Vehicles per square kilometre	City council
Number of incidents caused by alcohol or illicit drug consumption on the public road	Local Police
Number of crimes and incidents produced at the remodelled zone (segregated by age and gender of those affected)	Local Police
Number of citizens of the neighbourhood La Solana receiving social benefits (segregated by age and gender).	City council
Number of applications for housing aids at the neighbourhood La Solana	City council
Number of people of SAB getting to work by bicycle, scooter, or ways other than the car	City council
Number of people that use the remodelled zone (segregated by age and sex)	City council
Percentage of the population undertaking physical activity at the remodelled zone (segregated by age and sex)	City council

ASPCAT: Public Health Agency of Catalonia; AQUAS: Catalan Health Evaluation and Quality Agency.
